# Editorial: Theoretical and computational insights into brain-based cognition

**DOI:** 10.3389/fncom.2026.1986598

**Published:** 2026-09-21

**Authors:** José Roberto Castilho Piqueira, Antonio C. Roque, Renato Teodoro Ramos

**Affiliations:** 1 University of São Paulo, São Paulo, Brazil; 2 University of Toronto, Toronto, ON, Canada

**Keywords:** brain dynamics, cognition, models and modeling, networks, neuron

Understanding how cognitive functions emerge from the activity of the brain remains one of the central challenges of contemporary neuroscience. Cognition depends on processes occurring across multiple spatial and temporal scales, from the dynamics of individual neurons and local circuits to coordinated activity among distributed brain regions. Theoretical and computational approaches are therefore essential not only for describing neural activity, but also for identifying principles that can connect these different levels of organization and ultimately relate neural dynamics to perception, memory, behavior, and other cognitive functions.

The Research Topic *Theoretical and computational insights into brain-based cognition* was conceived to bring together complementary perspectives on this problem. The five contributions illustrate the diversity of approaches currently available, ranging from statistical analysis of large-scale electrophysiological recordings to mathematical descriptions of neuronal dynamics and broader conceptual frameworks concerning neural communication and emergence. Despite their methodological diversity, the articles share a common question: how can patterns of neural activity and interaction across scales give rise to organized brain function?

Two contributions address this question directly through the analysis of large-scale neural recordings. Bong et al., in *Cross-population amplitude coupling in high-dimensional oscillatory neural time series*, develop a statistical framework for identifying time-varying relationships between oscillatory activity recorded simultaneously from different brain regions. Rather than reducing multichannel recordings to single regional signals, their approach extracts latent components whose amplitude fluctuations reveal inter-regional interactions and their lead-lag structure. Applied to local field potentials from prefrontal cortex and visual area V4 during a memory task, the method provides a way of characterizing dynamic communication between high-dimensional neuronal populations. Importantly, the approach can identify amplitude-based relationships even in the absence of phase locking or coherence, thereby complementing more conventional measures of oscillatory coordination.

Olarinre et al., in *Relative timing and coupling of neural population bursts in large-scale recordings from multiple neuron populations*, approach inter-regional coordination from the perspective of transient population activity. Using large-scale Neuropixels recordings from multiple visual cortical areas and the thalamus, they develop a computationally efficient framework for estimating, on individual trials, the timing of stimulus-evoked population bursts and correlations between their timing across regions. Their results reveal both regularities consistent with the known organization of the visual system and substantial animal-to-animal variability. In particular, correlations among cortical areas tend to be stronger than cortico-thalamic correlations. Together, these first two studies emphasize that cognition-related neural coordination cannot be understood solely from average activity: temporal variability, population structure, and dynamically changing relationships between brain regions contain important information.

A complementary perspective is offered by Baker and Cariani in *A proposal for realizing cognitive functions using traveling waves, phase-locked and synchronized patterns, holography, neural mixing, and single sideband communications*. Rather than beginning with a particular dataset, the authors develop a broad hypothesis concerning how information might be encoded, distributed, transformed, and communicated in neural systems. Their framework combines traveling waves, temporal spike patterns, phase relationships, synchronization, recurrent networks, and distributed representations with concepts drawn from wave physics, holography, and communication theory. In this view, oscillations and traveling waves are not merely correlates of cognition but potential mechanisms for coordinating distributed neuronal populations and carrying temporally structured information. The proposal is deliberately integrative and generates hypotheses that can be confronted with increasingly detailed measurements of spatiotemporal neural dynamics.

The question of how descriptions at different scales can be connected mathematically is central to Çetin et al. in *Deterministic, stochastic, and mean-field PDE models in neuroscience*. Their review organizes differential-equation-based approaches into interconnected deterministic, stochastic, and population-level descriptions. Ordinary differential equations provide mechanistic descriptions ranging from single-neuron models to neural-mass and whole-brain systems; stochastic formulations incorporate variability intrinsic to neuronal activity; and mean-field partial differential equations describe collective dynamics in large neuronal populations. Viewed together, these approaches provide mathematical bridges between microscopic neuronal mechanisms and mesoscopic or macroscopic phenomena such as oscillations, synchronization, multistability, and pattern formation. The authors also highlight an important future challenge: integrating models across scales more systematically and connecting them more tightly with experimental data and modern methods of inference and machine learning.

Finally, Oruro et al., in *New directions for complex systems in contemporary neuroscience: a morphodynamic and emergent function approach*, broaden the multiscale perspective by emphasizing that neuronal morphology itself should be regarded as a dynamic level of organization rather than simply as a fixed substrate on which neural computation occurs. Their framework connects neuronal form, circuit organization, behavior, and emergent function, drawing examples from development, mother-infant interactions, epilepsy, and neuropsychiatric conditions. This perspective highlights a fundamental feature of brain-based cognition: causal relationships across levels are potentially reciprocal, with cellular and morphological processes shaping network dynamics and behavior while higher-level interactions and experience can, in turn, influence lower-level organization. Computational modeling offers an important means of investigating these cross-scale relationships.

Taken together, the contributions to this Research Topic illustrate that there is unlikely to be a single privileged scale or methodology for understanding brain-based cognition. Statistical methods are needed to extract meaningful interactions from increasingly high-dimensional recordings; dynamical models provide mechanistic links between neuronal and population activity; concepts from physics and information processing suggest possible organizing principles for neural communication; and complex-systems approaches emphasize emergence and reciprocal interactions across levels of organization. The challenge ahead is to bring these perspectives into closer contact with one another and with experimental data.

In this sense, the diversity of the five contributions is itself informative. Progress toward an explanation of cognition will depend not only on increasingly detailed measurements or increasingly sophisticated models, but on our ability to connect *structure, dynamics, information*, and *function* across scales. The articles collected here provide complementary steps toward that broader theoretical and computational synthesis.

